# Dawn of a new era in industrial photochemistry: the scale-up of micro- and mesostructured photoreactors

**DOI:** 10.3762/bjoc.16.202

**Published:** 2020-10-08

**Authors:** Emine Kayahan, Mathias Jacobs, Leen Braeken, Leen CJ Thomassen, Simon Kuhn, Tom van Gerven, M Enis Leblebici

**Affiliations:** 1 Center for Industrial Process Technology, Department of Chemical Engineering, KU Leuven, Diepenbeek, Belgium; 2 Process Engineering for Sustainable Systems, Department of Chemical Engineering, KU Leuven, Leuven, Belgium

**Keywords:** microreactor, microreactor scale-up, monolith reactors, packed bed reactor, photoreactor scale-up

## Abstract

Photochemical activation routes are gaining the attention of the scientific community since they can offer an alternative to the traditional chemical industry that mainly utilizes thermochemical activation of molecules. Photoreactions are fast and selective, which would potentially reduce the downstream costs significantly if the process is optimized properly. With the transition towards green chemistry, the traditional batch photoreactor operation is becoming abundant in this field. Process intensification efforts led to micro- and mesostructured flow photoreactors. In this work, we are reviewing structured photoreactors by elaborating on the bottleneck of this field: the development of an efficient scale-up strategy. In line with this, micro- and mesostructured bench-scale photoreactors were evaluated based on a new benchmark called photochemical space time yield (mol·day^−1^·kW^−1^), which takes into account the energy efficiency of the photoreactors. It was manifested that along with the selection of the photoreactor dimensions and an appropriate light source, optimization of the process conditions, such as the residence time and the concentration of the photoactive molecule is also crucial for an efficient photoreactor operation. In this paper, we are aiming to give a comprehensive understanding for scale-up strategies by benchmarking selected photoreactors and by discussing transport phenomena in several other photoreactors.

## Review

### Introduction

In the traditional chemical industry, thermochemical activation routes are mostly preferred. Light can also activate some molecules, which leads to fast and selective reaction pathways. Photochemistry spans a number of reactions. For organic chemistry, 8000 photoreactions have been listed since 1975 [1]. Despite the huge portfolio, there is a lack of industrial applications of photochemistry. Van Gerven et al. listed five industrial applications of photochemistry in commercial wastewater treatment installations [2]. In addition, artemisinin, which is a drug to treat malaria, was produced in an industrial-scale photoreactor in a Sanofi production facility in Italy [3]. Furthermore, the production of some fine chemicals, such as ε-caprolactam, rose oxide, and vitamin D on an industrial scale has already been proven to be successful [4]. These examples show that photochemistry is a viable alternative to conventional chemistry approaches. In addition to specialty chemicals and wastewater treatment, photochemical pathways can also be used for methanol production [5], N_2_ fixation [6], and CO_2_ sequestration [7]. Still, photochemistry has not been exploited much in industry since the distribution of light inside a photoreactor brings a lot of complexity to the reactor design. Unlike thermochemical reactions, it is not feasible to scale up photoreactions by increasing the dimensions of the reactor due to the exponential attenuation of light. Nonuniform light distribution often leads to a lower selectivity and longer reaction times, which in turn lowers the productivity [8–9]. Since photoreactions are intrinsically quite fast, mass transfer limitations should also be taken into account while designing multiphase photoreactors, and this complicates the design even further [10].

Photoreactions are typically performed in batch reactors. With the process intensification efforts towards green chemistry approaches, continuous flow technologies, micro- and mesostructured flow photoreactors having emerged as alternatives to batch operation. Due to the small characteristic length of microreactors, more uniform light distribution could be obtained. The elimination of overirradiation or dark zones results in less side product formation. Mass transfer limitations can be alleviated in these photoreactors with the help of various catalyst structures, beads, static mixers, and/or Taylor flow [4,11]. In addition, these reactors allow safer operation since it is easier to control overheating and the handling of hazardous chemicals in smaller volumes [4,11]. In addition to photomicroreactors, micro- and mesostructures in larger-scale photoreactors, such as packed beds and monoliths coated with catalysts can also enhance the mass transfer and operate at microreactor reaction rates. Together with photomicroreactors, these structured reactors are opening a new era in photochemistry by providing a platform for enhancing the photoreaction rates and easier scale-up.

One of the hurdles in scaling up of photoreactors is the lack of a consensus on a benchmark to compare different scales and geometries. The simplest benchmark is the apparent rate constant *k*_app_ [12–15] shown in Equation 1.

[1] $$k_{\text{app}}\;[{\text{s}}^{-1}]=\frac{c_{0}\;[\text{mol}\cdot {\text{m}}^{-3}]-c\;[\text{mol}\cdot {\text{m}}^{-3}]}{c\;[\text{mol}\cdot {\text{m}}^{-3}]\cdot \tau \;[\text{s}]}$$

where *c*_0_ is the initial concentration, *c* the concentration at the end of the reaction, and τ the residence time.

The comparison of the reaction rate constants for different reactor types, such as a photomicroreactor and a batch photoreactor are often used to point out how much the reactor design could change the performance of photoreactions. Takei et al. reported a reaction rate that is 70 times higher in a photomicroreactor compared to a batch cuvette, with the same yield for the synthesis of ʟ-pipecolinic acid [16]. The rate of methylene blue reduction increased more than 150 times in a photomicroreactor compared to a batch system [17]. Still, it should be kept in mind that such comparisons are often strongly biased due to the difference in the photons absorbed in different reactor geometries and the possible difference in mass transfer limitations. Another benchmark is the quantum yield ϕ*.* This value is defined by the IUPAC as the number of defined events occurring per photon absorbed by the system. This is shown in Equation 2.

[2] $$\phi \;[\frac{\text{mol}}{\text{einstein}}]=\frac{\text{number of reactant consumed or product formed [mol]}}{\text{amount of photons absorbed [einstein]}}$$

The photonic efficiency ξ (Equation 3) is defined by the IUPAC as the ratio of the reaction rate to the rate of incident photons within a defined wavelength interval [18]. This benchmark expresses the light efficiency. However, it does not provide information about the productivity.

[3] $$\zeta =\frac{r\text{ [mol}\cdot {\text{s}}^{-1}\text{]}}{\varphi \text{ [einstein}\cdot {\text{s}}^{-1}\text{]}}$$

where *r* is the reaction rate and φ is the rate of incident photons.

The aforementioned benchmarks focus on the efficiency of the photochemical process and do not consider the photon transport to the reactor, energy utilization, and throughput, and thus they are not suitable for comparing the scaled-up photoreactors. The space time yield (STY), as shown in Equation 4, is the amount of product produced per unit of time and reactor volume and is often used to compare scaled-up (nonphoto)reactors [19]. However, this benchmark does not include the energy consumption of the lamp utilized to provide the necessary energy. In a study by Leblebici et al., a new benchmark, the photochemical space time yield (PSTY), was proposed and used to compare different photoreactors, as shown in Equation 5. This benchmark relates the productivity to the energy efficiency. Therefore, it is commonly applied in recent studies to assess the scalability of the process [15,20–22].

[4] $$\text{STY [}\frac{\text{mol}}{{\text{m}}^{\text{3}}\cdot \text{s}}\text{] =}\frac{n\text{ [mol]}}{V_{\text{reactor}}\;[{\text{m}}^{3}]\cdot t\text{ [s]}}=\frac{c_{\text{0}}\text{ [mol}\cdot {\text{m}}^{-3}]\cdot \chi_{\text{a}}}{\tau \text{ [s]}}$$

[5] $$\text{PSTY [}\frac{\text{mol}}{\text{kW}\cdot \text{day}}]=\frac{\text{STY [mol}\cdot {\text{m}}^{-3}\cdot {\text{s}}^{-1}]}{P\text{ [kW]}/V_{\text{reactor}}{\text{ [m}}^{\text{3}}\text{]}}$$

where STY is the space time yield, *n* is the amount of product produced*, t* is the time, *V*_reactor_ is the reactor volume, *c*_0_ is the starting concentration, χ_a_ is the conversion, τ is the residence time, PSTY is the photocatalytic space time yield, and *P* is the lamp power.

In this paper, together with photomicroreactors, larger-scale photoreactors that contain micro- and mesostructures will be discussed since the performance and the problems related to scaling up are comparable. Our aim is to give an overview on the scale-up of photochemical reactions by discussing transport phenomena and technical challenges. In addition, we are assessing several high-throughput structured photoreactors using the PSTY to reveal the successful scale-up strategies in literature.

### Design considerations

**Figure 1:**
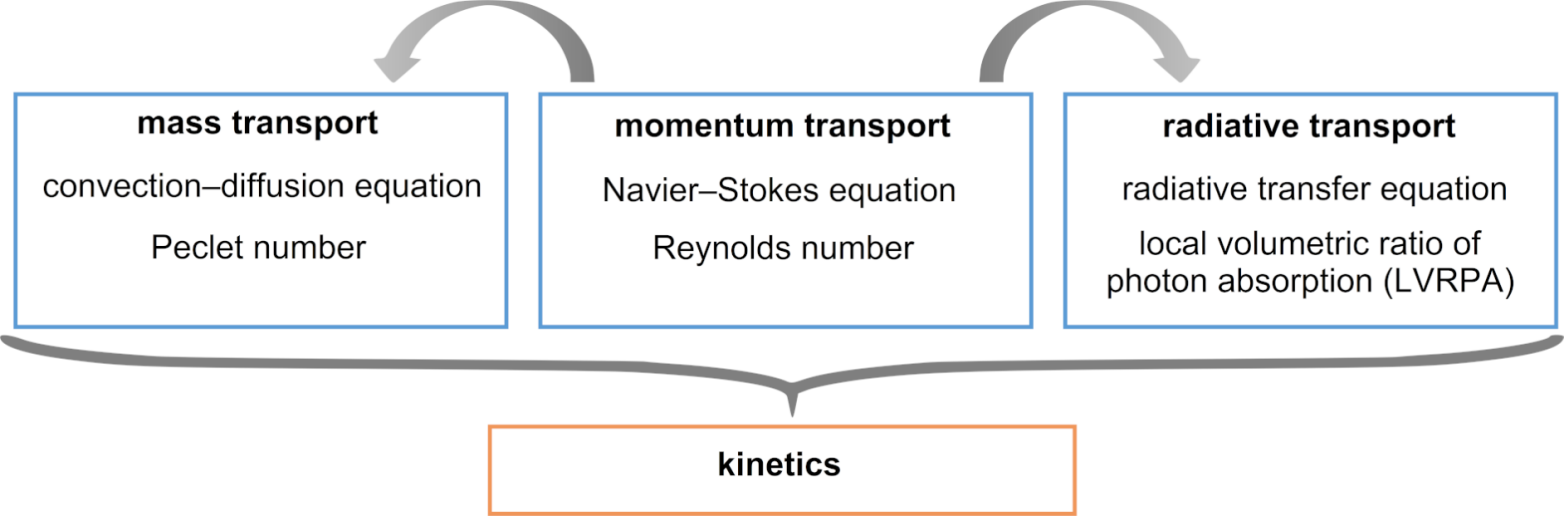
The momentum transport affects the mass transfer and the light field. All transport phenomena need to be coupled to compute the kinetics.

The complexity of the photochemical processes leads to significant challenges in the photoreactor design. In addition to the general reactor design considerations, such as the selection of a suitable reactor material and geometry, the selection of a proper light source needs to be considered. The reactor material should be inert to the reaction medium and transparent to the wavelength range that drives the photochemical reaction. Ideally, the light source should emit light only in the wavelength range of interest and should have a high energy efficiency. In addition, the solvent should not absorb light strongly nor be a quencher of the photoreaction. In order to avoid excessive heating of the light source, cooling systems might be necessary. The distribution of light inside the reactor brings a lot of complexity into the design. Since most photoactive molecules are strong absorbers of light, light usually decays within a few millimeters inside a photoreactor, which limits the dimensions of the photoreactors. As a result, many photoreactor units are usually required to achieve the desired throughput. In order to obtain similar reaction conditions, the flow needs to be distributed to all photoreactor units uniformly. In heterogeneous reactions, the flow field also affects the mass transport significantly. Structures such as beads or monoliths are frequently used to enhance the mass transfer, which in turn affects the flow field. If the photoactive molecule is the reactant, it depletes as the reaction proceeds. This introduces more variation to the light field, depending on the degree of mixing and the reaction rate. Due to the interplay of different transport phenomena, a mathematical description is often needed for a proper reactor design. The flow field (momentum transport), mass transport, and light field (radiative transport) need to be coupled to compute the reaction kinetics (Figure 1) [11].

**Figure 2:**
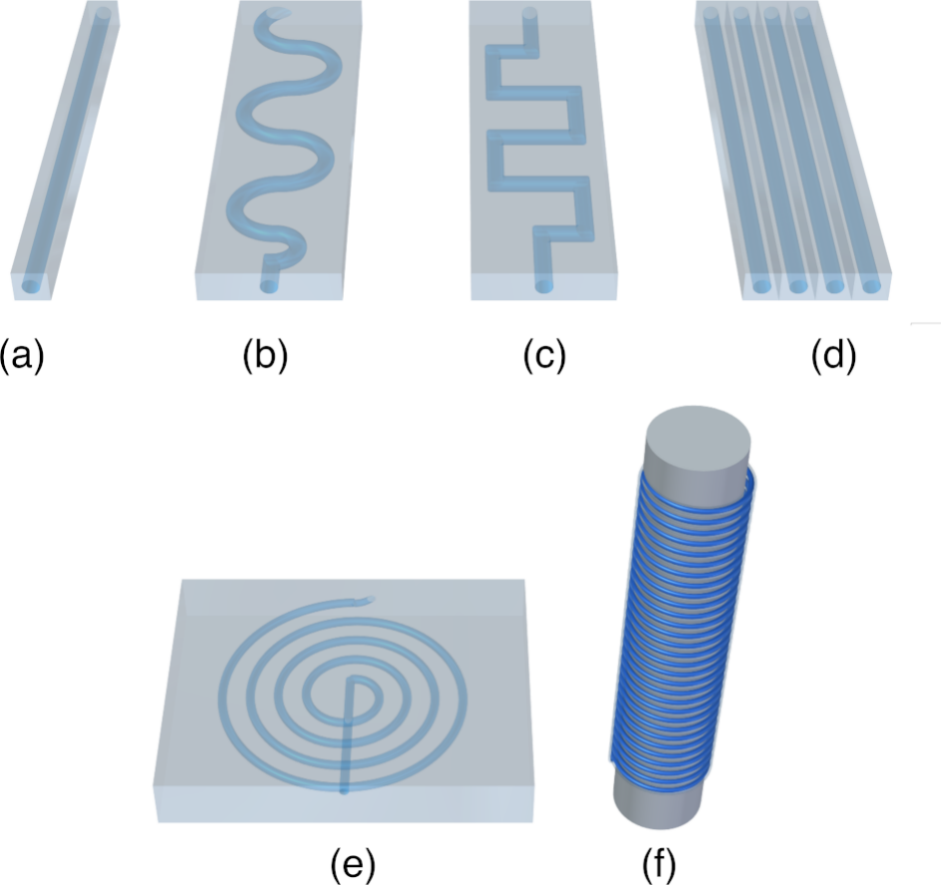
Common photomicroreactor designs: (a) Straight channel, (b) serpentine channel, (c) square serpentine channel, (d) planar microchannel array, (e) spiral-shaped microreactor, and (f) capillary photomicroreactor.

Microstructured chips used in photochemistry have various channel geometries, such as straight line, serpentine, square serpentine, and spiral, as shown in Figure 2. Serpentine and square serpentine are used to increase the residence time and for mixing. Microcapillaries (Figure 2f) wrapped around a light cylinder are also commonly used in photochemistry.

While designing microreactors, it is important to distinguish different applications. The lab-on-a-chip concept has enabled researchers to work on intrinsic kinetics. Intrinsic data acquisition is crucial while designing and operating large-scale reactors. Several microreactors, such as a spiral channel microreactor carved on a flat aluminum plate [23], a capillary tube [24], a microchannel cast, and cured on polydimethylsiloxane (PDMS) slab [25] or a rectangular slab micromachined on teflon [26] were used to determine the intrinsic kinetics of different photochemical reactions. Such demonstrations suggest that if they are scaled up properly, microreactors could be operated with intrinsic kinetics. This would reduce the reactor size and downstream the processing costs while increasing the safety. The design considerations for microreactors that aim at large-scale chemical synthesis are significantly different from the lab-on-a-chip concept. While gathering intrinsic kinetic data, energy efficiency is not a concern. Optimal photon flux should be provided to make sure that the reactor is operated without light transfer limitations. In addition, the utilization of one microchannel or a capillary tube would be enough as long as there is adequate mixing to overcome mass transfer limitations in multiphase reaction systems. On the other hand, studies aiming at the scale-up of microreactors should consider the distribution of the flow and light to all units of the microreactors, as well as the energy efficiency. A systematic scale-up strategy would be to increase the concentration of the chemicals and the reactor dimensions by keeping the yield constant while assessing the energy efficiency of the photoreactor by using benchmarks, such as PSTY. Then, the best reactor dimensions and operational parameters could be chosen. The PSTY of several photoreactor designs will be discussed in the next section.

### Scaling up and energy efficiency

Photoreactors cannot be scaled up by the conventional strategy of enlarging the dimensions due to the light attenuation effect. The heterogeneous flow dynamics and the light field complicate the design further. As a result, photochemical processes are hard to predict and to scale up. Several large-scale slurry reactor designs for multiphase reactions, such as the fountain reactor [27], the fluidized bed reactor [28], and spinning disc reactors [29–30] have been utilized in the field of photochemistry. Although such large-scale slurry reactors can improve the mass transfer, distributing light properly in a large-scale slurry reactor remains a challenge. The rapid attenuation of light inside photoreactors limits the dimensions of photoreactors. As a result, microstructured reactors are gaining more attention from researchers in this field. Leblebici et al. compared twelve commonly used photoreactor designs [22]. The microstructured reactor [25] resulted in the highest STY but the lowest PSTY due to the high power consumption of the light source [22]. In that work, a 120 W UV light source was used to illuminate a 1.5 µL reactor [25]. However, through the selection of a proper light source along with a systematic scale-up strategy by increasing the reactor dimensions and the concentration of the absorbing species gradually, microstructured photoreactors have the potential to achieve the highest PSTYs. It is also important to note that the PSTY does not account for the quantum yield. Therefore, the molecular and electronic features of the reactants, which could significantly affect the PSTY value, are not accounted for when comparing different reactions. As such, it is hard to make global conclusions just by looking at the PSTY value, especially when intermolecular and intramolecular reactions or homogenous and heterogeneous reactions are compared. Since the quantum yield of most photochemical reactions is not known, the PSTY still gives a good comparison between different reactor geometries. The PSTY especially gains importance when characterizing and deciding on the operational conditions of a photoreactor when a specific photoreaction is to be scaled up.

The flow and light need to be distributed properly to all units of the microstructured reactors while scaling up in order to ensure the same reaction conditions everywhere. For multiphase reactions, mass transfer issues need to be tackled with the design as well. Microreactors could be scaled up by numbering up the channels, which is also referred to as scaling out, or scaled up by enlarging the dimensions of the microchannels. Another way of scaling up reactors, which can work with intrinsic kinetics, is to create micro- or mesostructures in large vessels. Translucent packed bed reactors and aerosol photoreactors are examples of such designs. Below, we will discuss several scale-up strategies applied in photochemistry.

**Figure 3:**
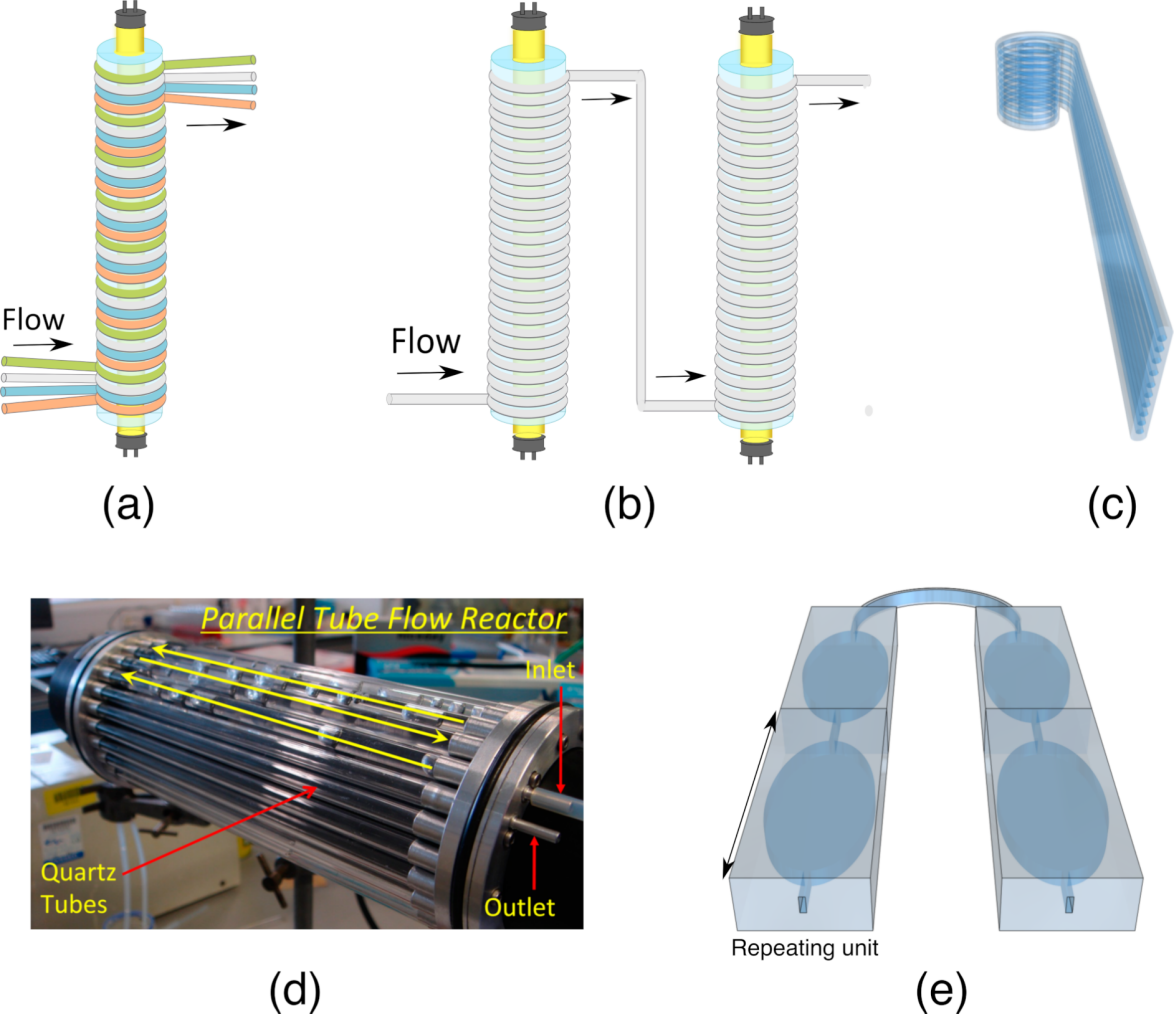
Benchmarked photoreactors: (a) Microcapillaries in parallel, (b) microcapillaries in series, (c) fluoropolymer microcapillary films, (d) firefly reactor (reprinted from [33], licensed under a Creative Commons Attribution 4.0 International license), and (e) internal and external numbering up of a microreactor unit. The image was drawn by the authors of this article to illustrate the microreactor structures reported in [34].

**Figure 4:**
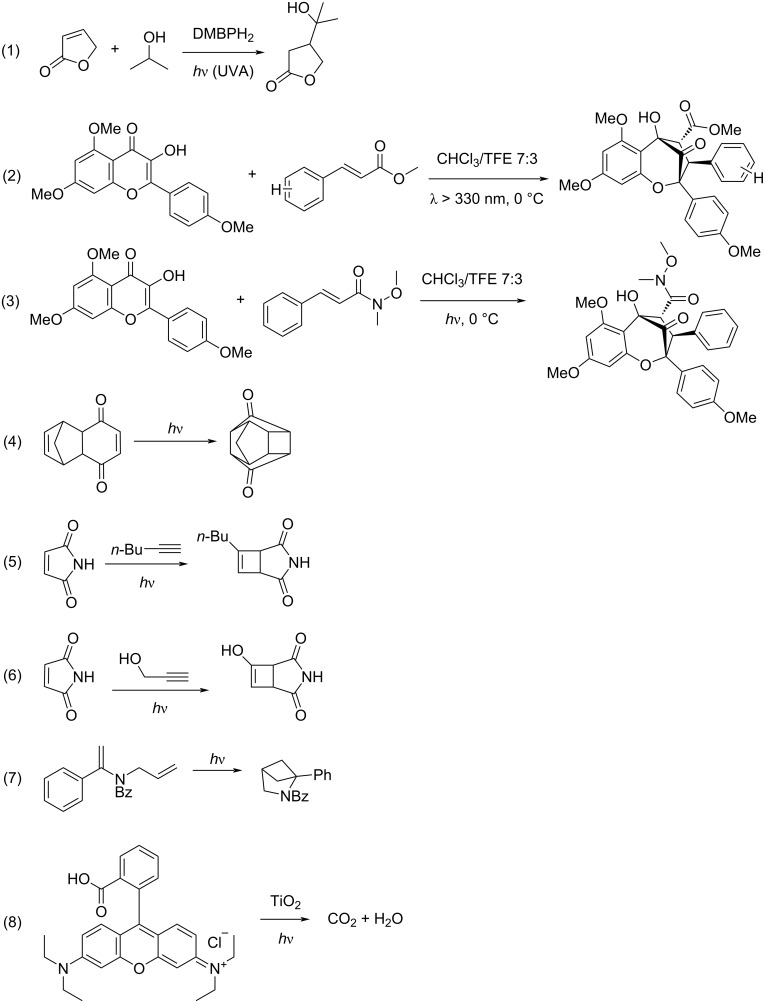
Photochemical reactions that are detailed in Table 1.

Most researchers that utilize photomicroreactors suggest that scaling up can be achieved by numbering up. One of the most used photomicroreactors are capillaries wrapped around a light source. Such microcapillaries can be scaled up by connecting them either in parallel or in series (Figure 3a and Figure 3b). In a previous study, five polymer-based microcapillaries with a length of 11.5 m and an inner diameter of 0.8 mm were wrapped around two Pyrex^®^ glass columns. UV lamps were placed inside the glass columns. A representative setup is given in Figure 3a. The addition of isopropanol to 2(5*H*)-furanone in the presence of the photosensitizer 4,4’-dimethoxy-benzophenone (DMBP) was studied. A single multisyringe pump was used to supply the reactants to the photomicroreactors. This kind of scaling up is called external numbering up since each reactor is fed directly by the pump. This numbering-up strategy allowed to run ten parallel microreactors at the same time. This microreactor setup used 30% less energy than the batch reactor, without the requirement of cooling [31]. The PSTY in this paper was calculated based on the same reaction being performed at the same time in ten parallel reactors (Table 1, entry 1). The reactions that are presented in Table 1 are shown in Figure 4. The PSTY was around 9.5 mol·day^−1^·kW^−1^ when the yield was 72%. To achieve almost complete conversion (94%), the residence time was increased twice. That decreased the PSTY to 6.26 mol·day^−1^·kW^−1^. Still, this microcapillary reactor outperformed all other reactors that were compared in this paper. This was because the large reactor volume (10 × 5 mL) was illuminated effectively by two low-power lamps (2 × 18 W). In addition, several photosensitizer and reactant concentrations were screened before the reaction was scaled up. With the selected photosensitizer concentration (10 mM), the light transmission through the 0.8 mm diameter microcapillary was around 65%. A higher DMBP concentration caused precipitations and resulted in a lower yield. Lower DMBP concentrations decreased the yield significantly, which decreased the PSTY values around 6-fold (data not shown). In another work, the same group reported the use of the same reaction in a commercial microchip (Micronit Microfluidics FC_R150.676.2) with an internal volume of 15 µm. This microchip was illuminated by 6 × 75 mW LEDs. The DMBP concentration was 6.7 mM, which resulted in around 95% light transmission through 150 µm channel depth of the microreactor [32]. This reactor had a PSTY of 0.57 mol·day^−1^·kW^−1^ (Table 1, entry 3). Although the same reaction and similar conversions were achieved in these two photomicroreactors [31–32], the PSTY was more than ten times lower in the microchip. This was potentially due to the difference in the volumes and the fraction of photons absorbed in two different photomicroreactors. These results illustrate that choosing a proper light source and adjusting the concentrations would improve the PSTY significantly, leading to an efficient scale-up of photomicroreactors.

**Table 1:** Comparison of different reactors in terms of PSTY. τ is the residence time and C is the concentration of the reactant. STY and PSTY are calculated based on Equation 4 and 5, respectively.

entry	reactor type	*V* (mL)	τ (min)	reaction (see Figure 4)	lamp power (W)	*c* (mM)	yield	STY (mmol·L^−1^ ·min^−1^)	PSTY (mol·day^−1^ ·kW^−1^)	Ref.

1	microcapillary photoreactors (Figure 3a)	50.00	5.0	1	36.0	33.3	0.71	4.72	9.43	[31]
2			10.0				0.94	3.13	6.26	
3	commercial microchip	0.02	2.5	1	0.5	33.3	0.89	11.87	0.57	[32]
4	microcapillary photoreactors (Figure 3b)	37.50	35.0	2	450.0	120.0	0.61	2.09	0.25	[35]
5		37.50	35.0	3	450.0	120.0	0.35	1.20	0.14	
6		12.00	20.0	3	300.0	120.0	0.32	1.92	0.11	
7		3.50	30.0	3	150.0	120.0	0.36	1.44	0.05	
8	fluoropolymer microcapillary films (Figure 3c)	0.15	0.5	removal of indigo carmine dye	8.0	0.2	0.70	0.30	0.01	[33]
9	firefly reactor (Figure 3d)	120.00	76.0	4	3000.0	1000.0	0.88	11.58	0.67	[36]
10			151.0	4	1500.0	500.0	0.89	2.95	0.34	
11			227.0	4	3000.0	1000.0	0.89	3.92	0.23	
12			76.0	5, 6	3000.0	100.0	0.65	0.86	0.05	
13			1342.0	7	3000.0	400.0	0.86	0.26	0.01	
14	microchip (Figure 3e)	1.00	960.0	8	0.2	0.012	0.78	9.74⋅10^−6^	7.01⋅10^−5^	[34]
15		0.80	960.0				0.85	1.06⋅10^−5^	6.12⋅10^−5^	
16		0.50	960.0				0.86	1.08⋅10^−5^	3.89⋅10^−5^	
17		0.01	480.0				0.98	2.46⋅10^−5^	1.77⋅10^−6^	
18		0.02	240.0				0.97	4.88⋅10^−5^	7.02⋅10^−6^	
19		0.04	120.0				0.98	9.76⋅10^−5^	2.81⋅10^−5^	
20		0.06	60.0				0.98	1.96⋅10^−4^	8.48⋅10^−5^	

In a previous study, a microcapillary reactor (1/6” OD, 3.5 mL reactor volume) that was wrapped around a metal halide UV lamp was scaled up by using several strategies together. Rather than splitting up the reaction medium into many photoreactors, the photoreactors were numbered up by adding them in series (Figure 3b). This allowed for a larger reactor volume with the same illumination. The throughput was increased by using a larger volumetric flow rate. Several [3 + 2] photocycloadditions were performed. While scaling up, the concentration of the reactants, the diameter of the tubes, and the number of the reactors were increased systematically. The throughput was increased around 20-fold by increasing the reactant concentration from 30 mM to 120 mM. The throughput was further increased by changing the diameter of the capillary from 1/6” to 1/8”. Finally, three reactors were connected in series, which resulted in a reactor volume of 37.5 ml. By using three reactors in series, with a larger capillary diameter, the throughput was increased from 0.14 g/L to 1.16 g/L. Similar yields were obtained in each step by adjusting the residence time [35]. The highest PSTY of this paper was calculated as 0.25 mol·day^−1^·kW^−1^ when a 37.5 mL reactor was used (Table 1, entry 4). The PSTY was decreased to 0.14 mol·day^−1^·kW^−1^ with a different reaction although the reaction conditions were the same (Table 1, entry 5). When smaller reactor volumes were used, the PSTY decreased further (Table 1, entries 5–7). The PSTY was expected to stay approximately the same when the reactors were connected in series since the lamp power and the throughput were increased at the same level. However, in Table 1, entries 5–7, in addition to using more reactors in series, the microcapillary diameter was also changed, which in turn changed the PSTY. The PSTY values of this paper were quite low, considering that the authors followed a systematic way of scaling up by utilizing several strategies together. The low PSTY might be associated with the challenging syntheses chosen in the paper. Previously, the synthesis of rocaglate was reported with an even higher lamp power (450 W) in a Pyrex^®^ test tube (16 × 100 mm) [37].

Fluoropolymer microcapillary films were suggested as another scale-up approach (Figure 3c). These microcapillary films were produced commercially by Lamina Dielectrics Ltd (UK). The internal diameters of the microcapillaries range from 10 µm to 1 mm. The microcapillary films are transparent to UV and visible light. Furthermore, they have a refractive index close to water (≈1.33), which makes them good candidates for photochemical reactors. In one study, those microcapillary films were used for the photodegradation of indigo carmine, diclofenac, and benzoylecgonine. The reactor volume was 0.15 mL [36]. The PSTY of the photodegradation of indigo carmine was calculated as 0.01 mol·day^−1^·kW^−1^ (Table 1, entry 8). In another study, those microcapillary films were used to synthesize ascaridole, an antimalarial drug, from α-terpinene by utilizing singlet oxygen. The permeability of the fluoropolymer film to oxygen was utilized to supply oxygen to microcapillaries. The system was suggested to be used in combination with dangerous gases to ensure safe operational conditions. With the fluoropolymer microcapillaries, space time yields 20 times larger than in the corresponding bulk synthesis were obtained [38]. Although it was suggested that fluoropolymer films could easily be scaled up by numbering up, how the flow was distributed to the microcapillaries was not explained in those studies [36,38].

In another scale-up study, quartz tubes were assembled in series and placed axially around a high-power UV lamp, as shown in Figure 3d. The reactor had an internal volume of 120 mL. The photoreactor was enclosed with an annular metal cavity that reflected UV radiation back to the reactor and served as UV protection. However, the metal cavity caused overheating. Therefore, a cooling jacket around the metal cavity was implemented in the design, along with a fan to blow air through the space between the lamp and the quartz photoreactor tubes. The design was called firefly reactor. Various [2 + 2] cycloadditions were performed in this reactor. Although similar amounts of starting material were used, the residence time varied significantly among different photoreactions to achieve the same yield [33]. The highest PSTY was calculated as 0.67 mol·day^−1^·kW^−1^ (Table 1, entry 9). This was the second-highest PSTY among the photoreactors compared in this paper. However, the PSTY ranged between 0.23 and 0.67 mol·day^−1^·kW^−1^ (Table 1, entries 9–11), depending on the residence time, lamp power, and the initial reactant concentration, although the same reaction was used, and similar yields were obtained. Therefore, a higher residence time reduced the PSTY around three times since the yield did not improve further (Table 1, entries 9 and 11). When different reactions were used, the same reactor resulted in a PSTY of 0.01 and 0.05 mol·day^−1^·kW^−1^ (Table 1, entries 9 and 11). This study demonstrated that the optimization of the process conditions and the kinetics of the selected reaction can lead to drastic changes in the PSTY.

de Sá et al. combined external and internal numbering up in meso- and microchemical reactors of various sizes (Figure 3e). Photocatalytic degradations of methylene blue, rhodamine B, and phenol with TiO_2_ were performed. Multiple microreactor units were connected to each other to increase the volume of the reactor while keeping the benefits of a microreactor. The reaction mixture was fully recycled when it left the reactor. Therefore, the reactor was operated as a batch reactor. Due to the small characteristic length of the reactor, there were less mass and photon transfer limitations compared to a conventional batch reactor [34]. The numbering-up was more beneficial in terms of STY and PSTY compared to increasing the dimensions, as shown in Table 1 entries 14–20. Increasing the volume from 0.5 mL to 1 mL by increasing the dimensions decreased the STY by 10%, whereas this increased the PSTY by 45%. When scaling up a reactor by increasing its dimensions, the surface-to-volume ratio decreases. This explains why the STY decreases: since the illuminated catalyst area per volume decreases, this leads to an increase in the mass transfer limitations and in turn to a lower apparent reaction rate and productivity. The PSTY increased since the same lamp was used to illuminate a larger reactor volume. Increasing the volume of the reactor by numbering up increased the STY and the PSTY by 97 and 98%, respectively. This is due to the increased total irradiated area while keeping the same surface-to-volume ratio.

Tree-like structures (bifurcation configuration) have been used to distribute the flow to many channels in various fields of research. Su et al. applied this numbering up approach to a capillary multiphase photoreactor by using the photocatalytic oxidation of thiols to disulfides as a model reaction [9]. Two, four, and eight photomicroreactors, each having a 0.5 mm internal diameter and a 0.95 mL volume were connected in parallel by using T-junctions. The fluid is distributed in a tree-like structure. A stable Taylor flow was obtained. The standard deviation of the flow distribution was less than 10%. The relative deviations of the liquid flow rate and the yield in each channel were found to be less than 4% [9]. Such a tree-like structure was also utilized in the scale-up of a luminescent solar photomicroreactors, which was fabricated via 3D printing [39]. Luminescent solar concentrators have been used in photovoltaic cell research for a couple of decades [40]. Noël’s group combined the use of luminescent solar concentrators with microfluidics to harvest solar radiation into a narrow wavelength region and derive photochemical reactions [41–43]. By distributing the flow with a tree-like structure, the luminescent solar photomicroreactor was operated with 32 channels, with less than 10% standard deviation in the flow distribution [39].

Enlarging one dimension while keeping the rest of the dimensions constant is another scale-up strategy. This strategy has been applied in commercial Corning Advanced-Flow™ reactors. Heart shapes provide good mixing for the liquid and gas phases and enhance the mass transfer while using the space on the microreactor chip efficiently. A photo of a Corning reactor is given in the Mass Transport section. Corning is offering reactors with different internal volumes. The lab-scale reactor has an internal volume of 2.6 mL, whereas the G1 and G3 photoreactors have an internal volume of 9 mL and 60 mL, respectively. All Corning reactors have the same heart-shaped static mixers [44]. The photocatalytic oxidation of methionine was performed in the Corning laboratory reactor. Two LED panels coupled with heat exchangers were placed on both sides of the reactor. Flow was supplied with an HPLC pump. Full conversion was achieved with a residence time ranging from 0.6 min to 1.4 min. This reaction could be scaled up using other Corning reactors. For example, if the same conditions could be maintained in a Corning G3 reactor, this reaction would have a productivity of 100 mol/day [45]. The power consumption of the LEDs was not reported in the paper. Therefore, the PSTY could not be calculated.

Packed-bed structures are often used for mass transfer enhancement. Once the channel size is adjusted, translucent packed structures can be used as an alternative scale-up strategy to the numbering-up of microreactors. Claes et al. adjusted the size of the beads in a catalytic packed bed reactor so that several microchannels were created among the beads. Glass beads were coated with a TiO_2_ photocatalyst. The photoreactor was illuminated with 192 LEDs that could provide 100 mW of power each. The distance between the LED board and the reactor was adjusted to give a uniform illumination, which resulted in a high light efficiency [15].

A novel approach to scale up microreactors is to use an aerosol photoreactor. In this reactor concept, micron-sized droplets are generated using a nebulizer. Each droplet works as a microreactor. The high surface area of the droplets enables fast mass transfer. As a result, aerosol photoreactors offer a great platform especially for gas–liquid or gas–liquid–solid photoreactions. When light hits a droplet, it is scattered. Therefore, the nature of the aerosol–light interactions enables good light distribution to all droplets. The reactor concept is easily scalable by simply increasing the number of nebulizers. An aerosol photoreactor called NebPhotOX has recently been operated several times and has been proved to work extremely efficient [46–48]. In that work, the liquid reactants were nebulized into a glass chamber that was wrapped with LED strips. A pneumatic nebulizer was used. The droplet diameter was reported by the nebulizer supplier as 6 µm for water-based solutions. The droplet size depends on the surface tension and viscosity of the liquid and the pressure of the gas. Therefore, it might be different for different solvents used in the photoreactor. Several singlet-oxygen-mediated photooxidation reactions were performed in the aerosol photoreactor. Singlet oxygen is a highly reactive state of oxygen, which can be formed by some photosensitizers or dyes when an appropriate light source is used. The authors performed an ene reaction of β-citronellol and Diels–Alder reactions of α-terpinene and (5-methylfuran-2-yl)methanol [46] as well as the synthesis of cyclopent-2-enones from furans [47] and the synthesis of diverse γ-lactam scaffolds [48]. Conversions larger than 90% were achieved for all reactions [46–48]. If the droplets are assumed to move with the gas, the residence time of the reactor would be around one minute. The PSTY of those papers could not be calculated, as the power consumption of the LEDs was not reported. Aerosol photoreactors are quite promising as they can solve the bottleneck of the photoreactor design, which is the distribution of the light efficiently in a large-scale reactor. Still, the light efficiency of aerosol reactors requires further work as it would depend on the droplet diameter, the aerosol number concentration, which is the total number of droplets per unit volume, and the light path of the reactor. The major drawback of the aerosol photoreactor are safety issues, especially for organic oxidation reactions. Spraying organics into air or oxygen could lead to explosions. Therefore, the lower and upper explosion limits for the several organic photooxidation reactions should be determined in order to ensure safe operating conditions.

### Mass transport

The mass transport phenomena can be quantified by the characteristic mixing time and the overall volumetric mass transfer coefficient. The characteristic mixing time in conventional reactors depends on many parameters, such as the diffusivity, kinematic viscosity, reactor volume, stirrer speed, etc. For a laminar flow in microstructured reactors, the characteristic mixing time can be calculated by the Einstein–Smoluchovski equation (Equation 6). Due to the short diffusion distances, complete mixing can be achieved rapidly. That is the reason why microreactors are suitable for reactions with a high intrinsic reaction rate [11].

[6] $$t_{\text{m}}=\frac{L^{2}}{D}$$

where *t*_m_ is the characteristic mixing time, *L* is the diffusion path, and *D* is the molecular diffusivity.

The relative importance of the characteristic mixing time in microreactors to the characteristic reaction time is given by the second Damköhler number (*Da*_II_, Equation 7). When *Da*_II_ is smaller than 1, the reaction is reaction-rate-limited. In this region, further mixing does not affect the reaction rate. When *Da*_II_ is around 1, the reaction is controlled by both the reaction rate and the mass transport. When *Da*_II_ is larger than 1, the reaction is mass-transport-limited. The residence time needs to be larger than the characteristic reaction time to achieve a complete conversion [11].

[7] $$Da_{\text{II}}=\frac{\text{reaction rate}}{\text{diffusive mass transfer rate}}=\frac{t_{\text{m}}}{t_{\text{r}}}$$

where *t*_r_ is the characteristic reaction time, which can be loosely defined as the inverse of the reaction rate constant.

Many photoreactions are heterogeneous, which means that the reaction requires the presence of at least two phases. Heterogeneous reactions require either a solid photocatalyst in a liquid medium or gas and liquid phases as the reactants. The mass transport and mixing gain extra importance in such systems. The mass transport is usually represented by the ratio of catalyst surface area to the reaction volume in photocatalytic systems. The photocatalyst could either be mixed with the reactants and fed into the reactor (slurry systems) or immobilized on a reactor surface. Slurry reactors remain the most preferred photoreactors due to the excellent contact between the catalyst and the reactants [2]. The ratio of the catalyst surface area to the reaction volume is quite high in slurry systems. However, the catalyst needs to be separated from the reaction medium in such systems, which adds complexity and additional costs to the overall process. It is possible to avoid the catalyst separation step with immobilized systems. Although they eliminate the catalyst separation step, the design of efficient immobilized systems is also quite challenging and requires complex mathematical models for the calculation of the internal mass and light transfer limitations. The catalyst thickness should be adjusted so that all of the catalyst is utilized. The optimum catalyst thickness would depend on the interplay of many parameters, such as the porosity of the catalyst and the support, the flow rate of the reactants, the light distribution throughout the catalyst layer, and the reaction rate. Leblebici et al. [49] showed that increasing the flow rate of the reactants did not increase the apparent reaction rate for phenol degradation in an immobilized photoreactor. Increasing the flow rate would result in a higher driving force for the mass transfer when there are no internal mass transfer limitations. As a result, it was concluded that the internal mass transfer limitation of the catalyst coating limited the reaction rate. Therefore, increasing the catalyst layer does not necessarily increase the apparent reaction rate [49].

**Figure 5:**
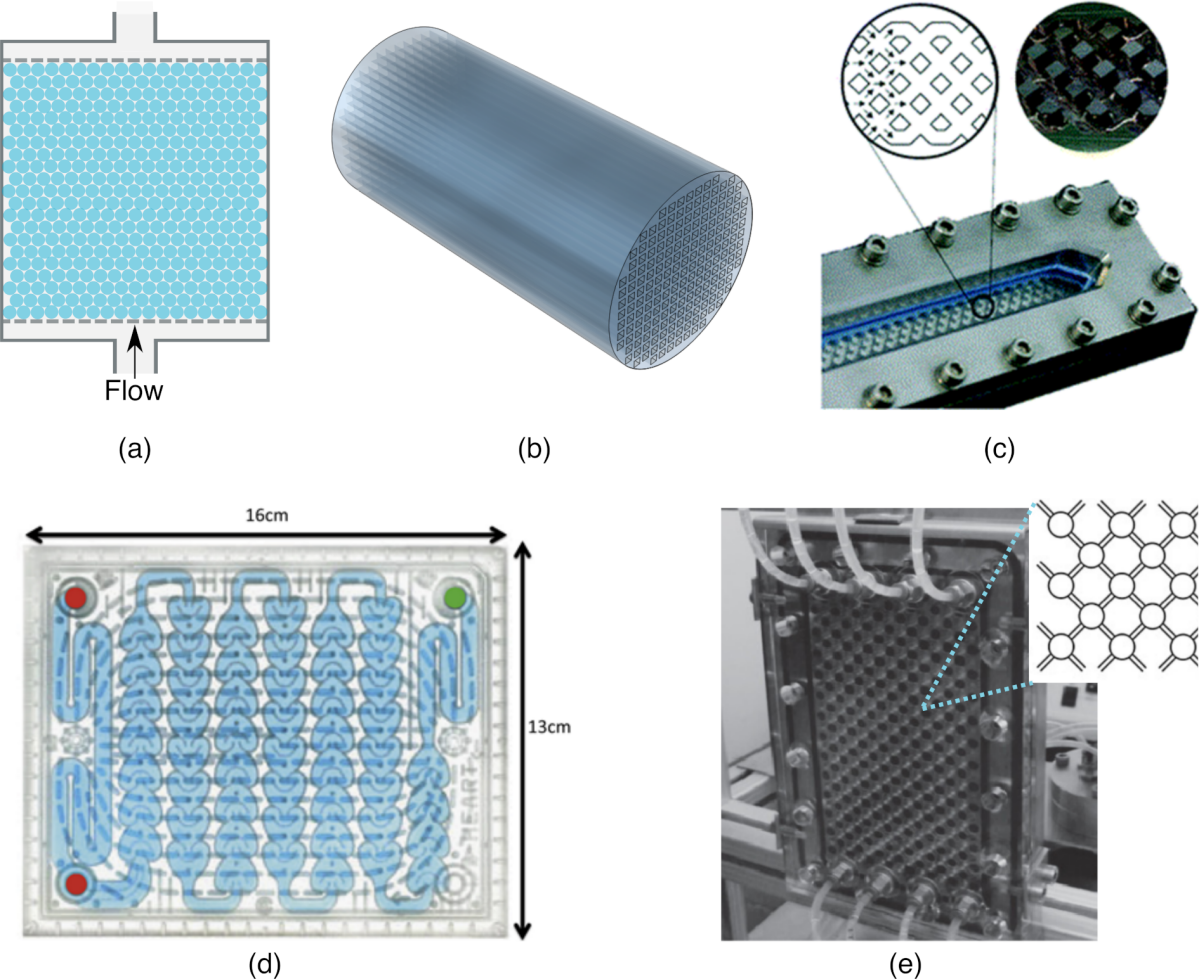
Structured reactors designed for enhancing the mass transfer: (a) Packed bed photoreactor, (b) monolith reactor, (c) HANU reactor (reprinted from [58] under a Creative Commons Attribution 3.0 Unported license), (d) commercial Corning Advanced-Flow^TM^ reactor (reprinted from [44] under a Creative Commons Attribution 3.0 Unported license), and (e) NETmix reactor (reprinted with permission from [59], copyright 2013 Elsevier).

Structures are commonly used to alleviate the mass transport effects. Catalysts are usually coated on beads [15,50–51], monoliths [52–54], or foams [55]. Beads coated with catalyst are often used in packed bed photoreactors. Schematic representations of a packed bed photoreactor and a monolith are shown in Figure 5a and Figure 5b, respectively. The performance of a capillary photomicroreactor packed with TiO_2_-coated glass beads were compared to a photomicroreactor having the walls coated with TiO_2_. The degradation of methylene blue was chosen as a model reaction. Complete conversion was achieved within 20 s when glass beads were used. The maximum space time yield of the packed bed photomicroreactor was found to be 2322 m^3^⋅day^−1^⋅m^−3^. On the other hand, in the wall-coated photomicroreactor, the maximum space time yield was found to be 356 m^3^⋅day^−1^⋅m^−3^. The significant improvement in the space time yield was attributed to the larger ratio of the catalyst surface area to the reactor volume in the packed bed reactor as well as the flow perturbation that increased the mass transfer rate. In addition, the packed bed photomicroreactor showed a good durability. The reactor performance dropped 17% after 6 h of operation and remained stable for the next 19.5 h [51]. Similarly, glass beads coated with photocatalysts were proved to increase the photoreaction rate twice in a luminescent solar concentrator (LSC) photomicroreactor with capillary channels [50]. Azam et al. studied monolithic honeycomb structures coated with La/TiO_2_ for hydrogen production. The photocatalytic activity was increased 1.8-fold in the monolithic photoreactor compared to the slurry photoreactor. The increased photocatalytic activity led to a 9-fold higher hydrogen production rate in the monolithic photoreactor. The main reasons for this increase was the high surface-area-to-volume ratio and better illumination of the monolithic photoreactor [56]. The monolithic photoreactor was shown to have a higher yield for the CO_2_ photoreduction compared to a cell-type reactor that had no channels. This was associated with a better illumination efficiency and a higher catalytic surface area [57]. There are several commercial photoreactors that have been designed with static mixers to enhance the mass transfer, such as the HANU photoreactor and Corning Advanced-Flow^TM^ reactors (Figure 5c and Figure 5d). The combination of an oscillatory flow with the static mixers of the HANU reactor (Figure 5c) enabled a stable suspension of the photocatalysts without clogging of the reactor channels [58]. Corning flow reactors are designed to provide good mixing for multiphase reactions with periodic heart-shaped static mixers (Figure 5d). Two-phase flow transitions from a Taylor flow to a stratified flow and further to a dispersed flow were investigated. The interfacial mass transfer and phase transitions were shown to be driven by fluid–structure interactions on the milli scale. Therefore, this reactor design has a straightforward scalability with the same mass transfer characteristics by keeping the periodic heart-shaped static mixers [44].

The NETmix reactor (shown in Figure 5e) was originally developed by Brito Lopes et al. [60]. The NETmix reactor was coated with a TiO_2_ photocatalyst for the removal of oxytetracycline (a pharmaceutical micropollutant) from urban wastewater. The effect of a catalyst-coated surface and the illumination mechanism were studied. When a larger surface area was coated with the photocatalysts, the photocatalyst reactivity was increased [61]. Marinho et al. compared the performance of the NETmix reactor with a monolithic reactor packed with translucent cellulose acetate monolithic structures. The front glass of the slab, the network of the channels, and the chambers imprinted in the back stainless steel slab of the NETmix reactor were coated with a thin-film photocatalyst (TiO_2_-P25) by using a spray system. The catalyst reactivity in the NETmix reactor was 70 times higher than for the monolithic photoreactor [62].

When the reactions involve a gas phase, a Taylor flow, also known as a slug flow, is commonly utilized in microstructured reactors. Su et al. studied the intrinsic kinetics of the photocatalytic oxidation of thiophenol to phenyl disulfide using a Taylor flow in a microreactor that consists of a capillary tube. By varying the flow rate of the gas and liquid phase and comparing the oxygen concentration in the liquid phase with the equilibrium concentration without a reaction, the authors showed that mass transfer limitations were overcome in the microreactor [24].

### Photon transport

A uniform light field is desired inside photoreactors. However, due to the exponential decay of the light intensity along the light path, having a uniform light field throughout the reactor usually means that light is wasted. On the other hand, if the utilization of all the light is desired, some of the reactors would be overilluminated, and the rest would be poorly illuminated. Reflective surfaces would eliminate this problem to some degree. Still, for an efficient photoreactor operation, the light source and intensity need to be properly coupled with the reactor geometry. While selecting the light source, the light spectrum and energy efficiency of the lamp and shape of the reactor should be considered. Ideally, the light source should emit light only at specific wavelengths that match the absorption spectrum of the photoactive molecule. In addition, the distance between the light source and the reactor should be taken into account while positioning the lamp. The irradiance pattern changes depending on the distance from the light source. At a long distance between the light source and the lamp, the irradiance decreases with the square root of the distance from a point light source according to the inverse square law (Equation 8).

[8] $$E\propto \frac{1}{d^{2}}$$

where *E* is the irradiance and *d* is the distance between the object and the light path.

In the near field, the irradiance can be modeled with ray tracing algorithms or it can be measured experimentally with a near field goniophotometer. The far field for LEDs is usually assumed to start at a distance that is five times larger than the dimensions of the light source. In reality, the distance for the far field can go up to twenty times larger than the dimensions of the light source [63–64]. As mentioned in the previous section, just adjusting the irradiance can lead to a PSTY two orders of magnitude higher. Ziegenbalg et al. pointed out the importance of the geometrical compatibility between the light source and the photomicroreactor by investigating a glass microreactor coupled with organic light-emitting diodes. The authors showed that 63% of the photons were lost due to the mismatch between the reactor channels and the light source, whereas 17% of the photons were lost due to the spectra mismatch [65]. While designing light sources, heat generated by the light sources should be considered as well. Excess heat can lead to damages of the whole setup. In addition, the spectrum and the energy efficiency of the lamp can be affected by the temperature. For instance, the LED junction temperature affects the optical output power and spectrum of the LEDs and as a result decrease the efficiency of the LEDs [66].

**Figure 6:**
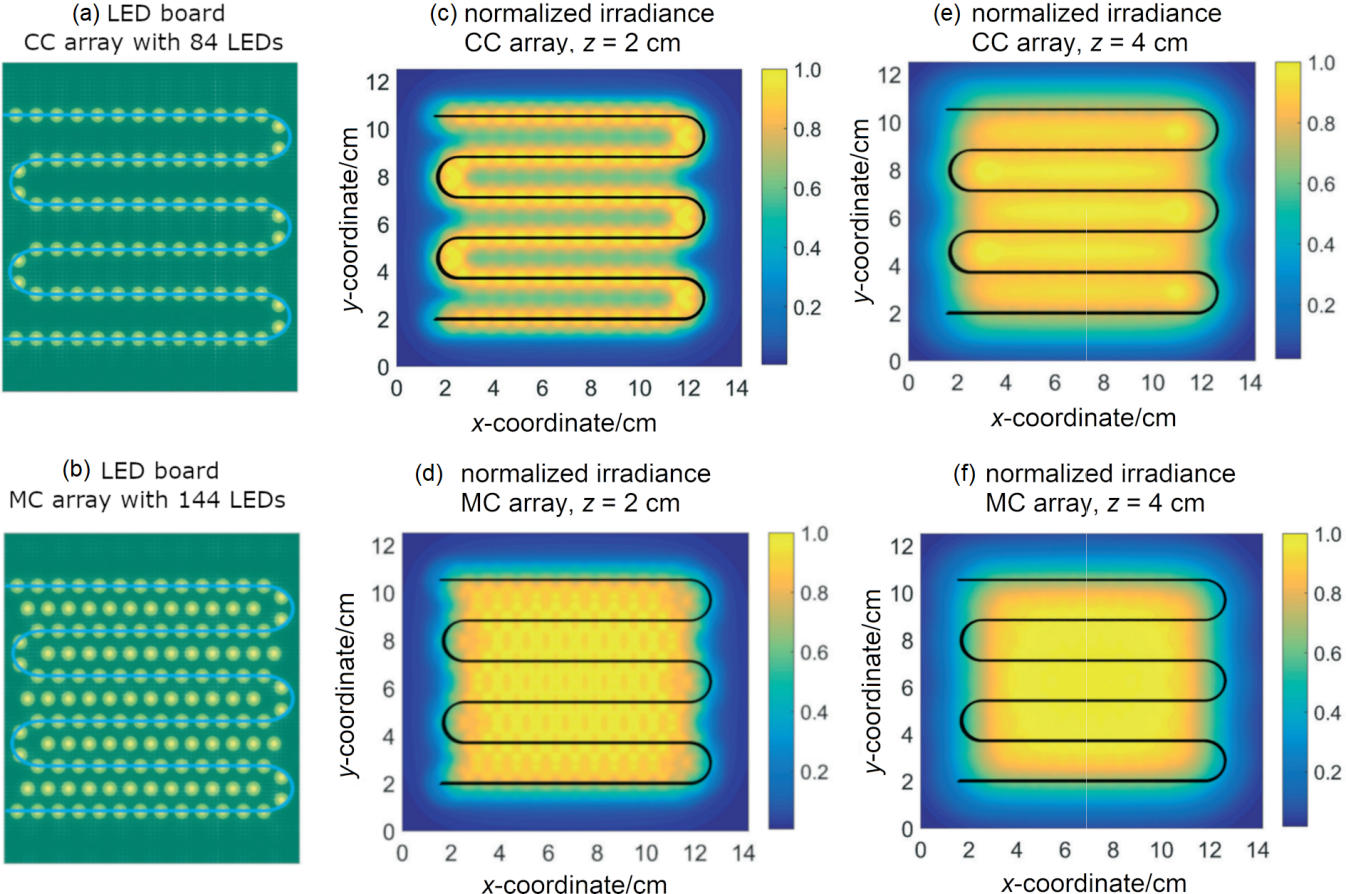
Comparison of the LED board designs of photomicroreactors: (a) CC array design, (b) MC array design. Normalized irradiance on a parallel plane placed at (c) *z* = 2 cm from a CC array, d) *z* = 2 cm from an MC array, e) *z* = 4 cm from a CC array, f) *z* = 4 cm from an MC array. The photomicroreactor channels are represented by the S-shaped blue and black lines. The photomicroreactor dimensions are not drawn to scale. Reprinted with permission from [63], copyright 2018 The Royal Society of Chemistry.

LEDs are usually preferred to illuminate microstructured photoreactors due to their small size, monochromaticity, and adjustable power. LEDs are also the most efficient light sources in the visible range [67]. Roibu et al. designed LED boards consisting of narrow-viewing-angle LEDs for an optimal coupling between the light source and the photomicroreactor. The design of the two LED boards, which were called the channel configuration (CC) and the matrix configuration (MC), is shown in Figure 6a and Figure 6b. The distance between the adjacent LEDs was 8 mm. The irradiance values at different distances *z* between the tip of the LED and the surface of the reactor was modeled with a ray tracing algorithm. The results were validated by experimental measurements. For these LED boards, the optimum *z* value where a uniform light distribution can be obtained on the surface of the photomicroreactor was found to be 1.5 cm. The normalized irradiance value when *z* was 2 cm and 4 cm, respectively, is shown in Figure 6c–f. The most uniform irradiance was obtained with the CC array when *z* was 2 cm. The irradiance at the outer edges was lower compared to the central sections for the MC array. The irradiance became more homogenous at the central sections when *z* was increased to 4 cm. However, the overall uniformity decreased as the irradiance at the edges decreased even more. When the LEDs were configured in the same shape as the reactor, the energy efficiency was the highest [63].

As mentioned in the previous section, structures such as beads or monolith supports are frequently used to increase the mass transfer in photoreactors. Zhao et al. studied beads made of glass, zircon, or steel in a capillary-based photomicroreactor. Opaque packing (steel) decreased the conversion by 5-15%, depending on the flow rate. A material with a higher refractive index increased the conversion [50]. This is in line with the observations in the work of Cambié et al. [43]. Ramos et al. also confirmed that the utilization of a transparent packed material increased the apparent reaction rate by a 4-fold compared to an iron packing material [68]. Therefore, a translucent material should be preferred in photoreactors.

The photon flux received by the absorbing species needs to be determined accurately in order to characterize photochemical reactors. The calculation of the local volumetric rate of photon (or energy) absorption (LVRPA or LVREA) is the main aspect of the modeling of photoreactors. LVRPA represents the amount of photons absorbed per unit time and per unit reactor volume at different locations in the reactor. Then, the reaction rate at a given location can be related to the light field by using the quantum yield (Equation 9), where *r* is the reaction rate (mol⋅m^−3^⋅s^−1^), ϕ is the quantum yield (mol⋅mol⋅photons^−1^), and LVRPA is local volumetric rate of photon absorption (mol⋅photon⋅m^−3^⋅s^−1^). In order to obtain the average reaction rate, both the concentration field and the light field inside the reactor need to be solved. The concentration field inside the reactor depends on the hydrodynamics. For ideal reactors (perfectly mixed or plug flow), the reaction rate would still be heterogeneous due to the attenuation of the light inside the reactor [4]. The light field in single phase for homogenous photoreactions can be easily predicted by the Beer–Lambert–Bouguer law (Equation 10).

[9] −r=ϕ LVRPA

[10] $$A=\log_{10}\frac{E}{E_{0}}=\varepsilon \cdot l\cdot C$$

where *A* is absorbance, *E*_0_ is the incident irradiance (W⋅m^−2^), *E* is the irradiance transmitted by the medium (W⋅m^−2^), *ε* is the absorptivity (m^2^⋅mol^−1^), *l* is the path length (m), and *C* is the concentration of the attenuating species (mol⋅m^−3^).

**Figure 7:**
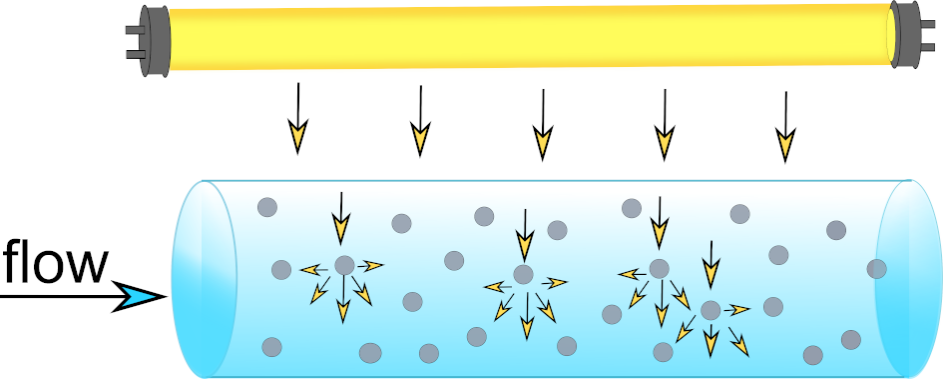
Illustration of the light scattering phenomenon inside a photocatalytic flow reactor.

Photoreactions are often heterogeneous, with solid particles in the reaction medium. In addition, some photoreactions contain a gas phase, which further complicates light field models. For heterogeneous photoreactions, the liquid reaction medium is usually transparent to light. Solid photocatalysts, on the other hand, are absorbing and scattering light. The scattering of light by a particle depends on the refractive index of the medium and the particle, the particle size, and the wavelength of light. The ratio of the diameter of the particle to the wavelength of the light is called the size parameter. The scattering phenomenon in a tubular reactor is depicted in Figure 7.

**Figure 8:**
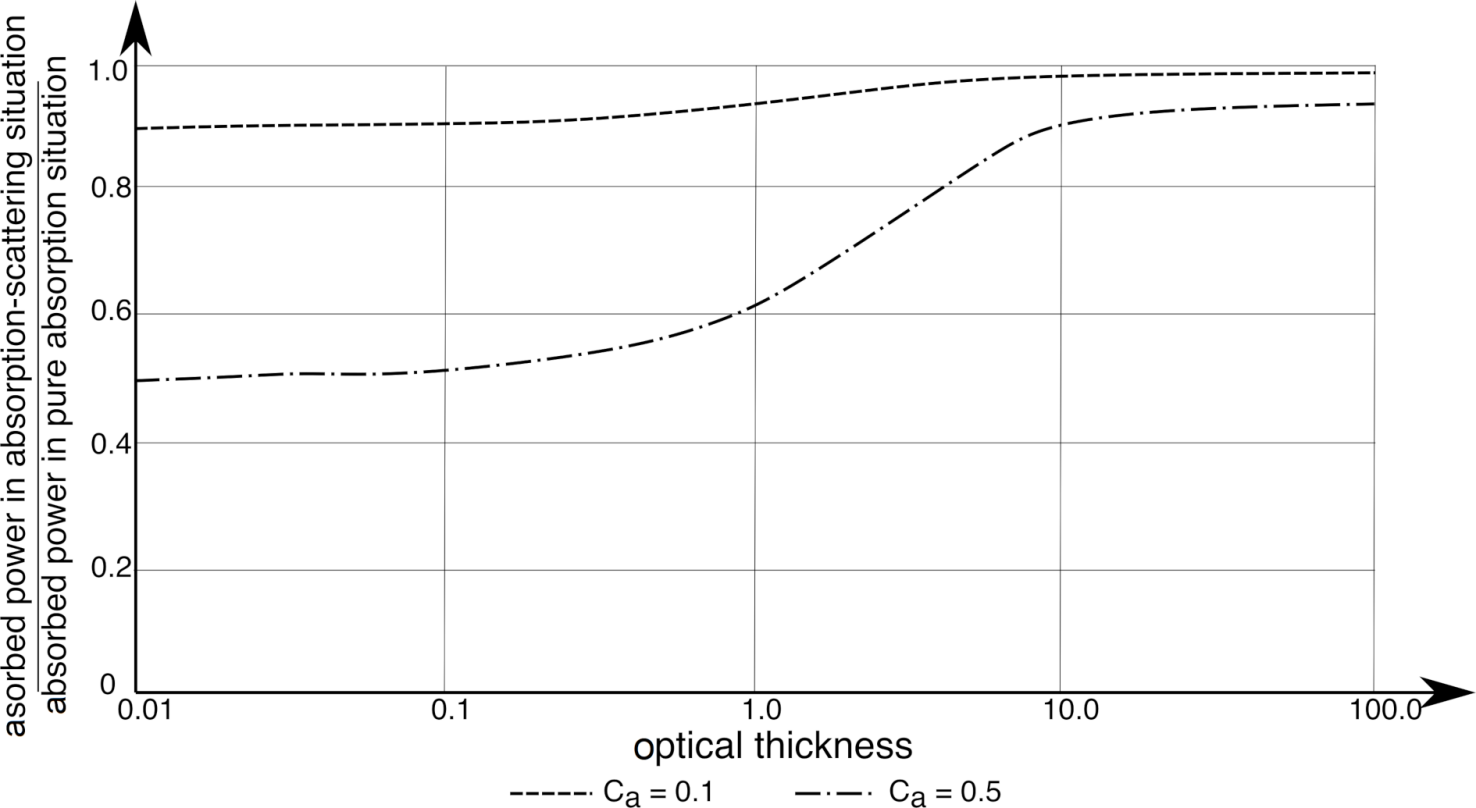
Efficiency of the absorption process in scattering situations with respect to pure absorption situations. The figure was redrawn from a paper by Spadoni et al. [69].

The efficiency of the absorption in scattering media with respect to pure absorption situations is shown in Figure 8. The optical thickness is the natural logarithm of the ratio of the incident to the transmitted irradiance. The efficiency of the absorption process approaches 1 at a large optical thickness. For an optically thick medium, the mean free path of a photon becomes so short that the medium can be considered as a continuum with a constant optical thickness [69].

The scattering by a single particle is described by Mie’s theory, who solved the Maxwell equations for a plane electromagnetic wave encountering a sphere. Mie’s theory gives exact analytical solutions for all size parameters. Still, Rayleigh scattering is preferred for small size parameters. For large size parameters, ray tracing, which is also called geometric optics, is less computationally demanding and therefore more frequently used in this region. Once the scattering profile of a photocatalyst particle is obtained, the radiative transfer equation still needs to be solved to obtain the LVRPA value. The integro-differential nature of the equation makes it quite hard to obtain an analytical solution. Therefore, approximations, such zero reflectance [70], two flux [71], and six flux [72] were suggested to model the slurry photocatalytic systems. Among the simplified models, two flux gives a simple and still accurate solution of the radiative transfer equation in a scattering medium. The model assumes that the particles are purely backscattering the photon. The six flux model assumes that photons flow the six routes of the coordinate system. Therefore, it is more accurate. Six flux is slightly more complex than the two flux approximation, but it is still an analytical equation that can be easily calculated if the single scattering albedo is known. If the absorbance of the photocatalyst is known, the single scattering albedo can be obtained by using online tools that solve Mie’s theory [73]. For solid photocatalysts, the absorbance is usually unknown. Therefore, the single scattering albedos for solid photocatalysts are usually obtained experimentally.

Scattering phenomena are seen in all dimensions while designing a photoreactor. Not only the structures in the photoreactors but also the reactor tubes would scatter light when a bundle of tubes is illuminated. In a previous study, three different configurations of tube bundles in a 20 mm tube photoreactor were studied. The tube bundle consisted of 31 tubes of a 3 mm outer diameter, 19 tubes of a 4 mm outer diameter, and 6 tubes of an 8 mm outer diameter. An array of 6 tubes resulted in the best performance. The authors concluded that the effectively illuminated surface area was of secondary importance in the design of the tube bundles [74]. Jacobs et al. used ray tracing to model a 3D-printed translucent monolith. The diameter (*D*), the number of channels, and the distance between the channels (*L*) were varied in the model. The optimal *L*/*D* ratio was found to be 2. Increasing the number of channels stacked upon each other up to 6 channels increased the amount of absorbed light and the PSTY, with the cost of increased inhomogeneous irradiation. Since translucent monoliths do not have the disadvantages of an optical fiber reactor, they showed an improvement of seven orders of magnitude in terms of the PSTY compared to internally illuminated monoliths.

Gas–liquid reactions in photomicroreactors use the Taylor flow, which is characterized by the regularly sized bubbles surrounded by liquid slugs. The light distribution is affected by the presence of bubbles in the Taylor flow. In a recent study, a photochemical reaction model was developed to optimize the performance of reactors that use a Taylor flow in a serpentine-shaped photomicroreactor. The photon flux per liquid volume was shown to increase exponentially with the amount of gas inside the channels. The conversion was significantly affected by the liquid distribution inside the channels rather than the light scattering or the liquid mixing. In that work, an empirical formula was suggested for the prediction of the optical path length in gas–liquid flows [75]. The photon transport, together with the hydrodynamics in the commercially available Corning G1 Advanced-Flow^TM^ reactor (G1 AFR) was studied. A Corning AFR was shown in the previous section in Figure 5d. It was concluded that the photon flux per unit volume and the hydrodynamics did not depend on the gas content for a wide range of flow conditions, which showed the high flexibility of the AFR. In that work, an empirical formula was suggested for the optical path length [76].

The utilization of solar energy for photoreactions can significantly reduce the cost of the process. In order to use solar energy effectively, the use of luminescent solar concentrators (LSC) together with flow photochemistry is suggested. LSCs are devices in which luminophores, such as fluorescent dyes or quantum dots, are dispersed in a glass or polymeric material to capture the sunlight. The luminophore absorbs sunlight and can emit a specific wavelength of light. LCSs have often been coupled with photovoltaics. Cambié et al. embedded a microstructured photoreactor in a luminophore. The reactor was benchmarked with a [4 + 2] cycloaddition. Monte Carlo ray tracing simulations showed that the aspect ratio of the channels, the relative height of the channels compared to the device thickness, and the number of channels per unit area were the most important design parameters [41]. In a recent work, the efficiency of the photomicroreactors embedded in LSCs was demonstrated for many homogenous and heterogeneous photoreactions, which span the entire visible spectrum. An autonomous control system that changed the residence time based on the fluctuations of the solar light intensity was successfully operated [43]. These operations of photomicroreactors embedded in LSCs are quite promising since they have the potential to reduce the energy costs significantly.

### Flow distribution and fabrication of microreactors

The homogenous distribution of fluids while numbering up microreactors is still one of the challenges when numbering up. A nonuniform flow distribution can cause a plethora of problems, such under- and overirradiation and a lower conversion. The maldistribution index (MI) is a common method to assess the flow distribution in internally scaled-up reactors [9,77–80]. It is defined as the relative standard deviation of the mass flow rate inside the microreactors or channels. This is shown in Equation 11.

[11] $$\text{MI}=\sqrt{\frac{1}{n-1}\sum_{i\text{ = 1}}^{\text{n}}{\left(\frac{q_{i}-\bar{q}}{\bar{q}}\right)}^{2}\cdot 100\%}$$

where *n* is the number of channels, *q*_i_ is the mass flow rate in a channel, and q̄ is the mean flow rate. A low MI indicates a uniform flow distribution.

**Figure 9:**
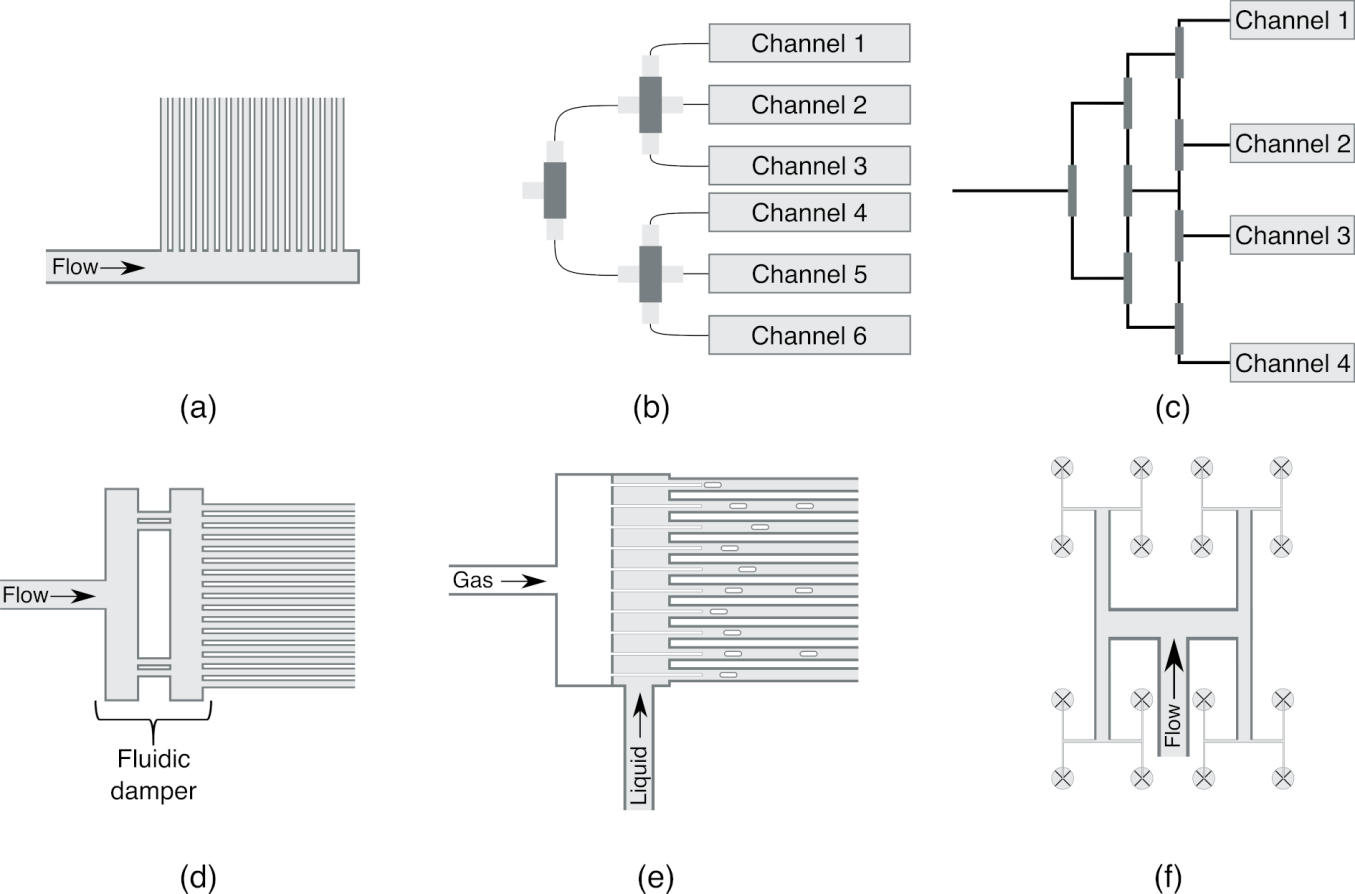
Different types of distributors: (a) Traditional or consecutive manifold, (b) bifurcation unit distributing flow to 6 microreactors (MR), (c) split-and-recombine, (d) distributor with fluidic damper, (e) needle distributor, and (f) fractal distributor (the channels are perpendicular to the plane of the paper and are depicted with an X).

Numerous distributors can be used to number up photochemical microreactors. These are not limited to the field of photochemistry, and inspiration can be found in the field of heat exchangers and other microreactors. These distributors are categorized and shown in Figure 9. When selecting a distributor to scale up (photo)chemical microreactors by numbering up, a careful consideration based on the flow rate as well as the amount of channels and phases that need to be distributed in the microreactors must be made.

The traditional manifolds, also known as consecutive manifolds, are one of the most used distributors for microreactor arrays due to the simplicity and well-known design rules. They are used for single-phase reactions at a low flow rate. Discrete methods are used to study the flow distribution and pressure drop in manifold systems. In these methods, a lattice network of resistances describes the system, which is analogous to electrical resistance networks. The flow rate is assumed to be linearly related to the pressure drop. The Darcy–Weisbach or Hagen–Poiseuille equation is used in combination with Kirchhoff's law to predict the flow rate in each channel [81–84]. A consecutive manifold is shown in Figure 9a. It is also possible to perform computational fluid dynamics (CFD) simulations to design a distributor. However, the resistance network models are usually preferred over CFD studies due to simplicity [85].

A bifurcation manifold is inspired by nature (e.g., trees, blood vessels, and lungs). The flow is divided by tree-like structures, as shown in Figure 9b. The same discrete methods of consecutive manifolds can be used to design a bifurcation distributor [83]. In contrast to the consecutive manifold, this distributor can be used to distribute the gas and liquid efficiently [9]. Furthermore, the homogeneity of the flow distribution does not change for different flow rates if all the channels have the same characteristic length [83]. However, when a part of the network clogs, all downstream microreactors will suffer from this. Nagaki et al. have developed a split-and-recombine bifurcation distributor to mitigate the aforementioned issue by recombining the flow after each level of distribution, as shown in Figure 9c [86].

When high flow rates are used in manifolds, the flow distribution will most likely not be uniform, due to the inertial force [87]. It is known that placing baffles or a fluidic damper, with protrusions or holes, increases the hydraulic resistance so that a uniform flow can be obtained. Park et al. developed a method to design distributors containing baffles to obtain a uniform flow while utilizing a high flow rate [87]. Furthermore, this design can be used when the system contains a large number of microreactors, which is not the case with bifurcation manifolds. This design type is shown in Figure 9d.

A different approach to distribute the fluid and gas over a stack of microreactors is to put needle-like structures inside the channels, as shown in Figure 9e. This distributor was able to distribute the flow over 357 channels with a MI lower than 3% [88–89]. Fractals are selfsimilar-repeating geometric structures that are invariant when scaling up or scaling down [90]. Mazur et al. obtained an MI of 1.63% when distributing a fluid with a fractal distributor over 64 channels at a Reynolds number of 10000, and thus making the fractal distributor an interesting option when picking a suitable distributor for the microreactor [91]. A method to optimize this type of distributor is presented in the work of Wang et al. They used the lattice Boltzmann method to optimize the shape of a fractal distributor to obtain a lower pressure drop [92]. The lattice Boltzmann method is an alternative to traditional CFD methods to calculate the flow field. A pressure drop reduction between 15.9% and 25.1% was obtained by shape optimizations, in which the T-junctions evolved into Y-junctions by the algorithm. The design of the fractal distributors is shown in Figure 9f. A comparison of the different distributors is shown in Table 2, and this Table can be used to select a suitable distributor.

**Table 2:** Comparison of different distributors.

type	phase	inlet liquid flow rate (mL/min)	Reynolds number inlet	number of microreactors fed	MI (%)	Ref.

consecutive manifold	liquid	0.01	0.1–10	10	0–16	[93]
consecutive manifold	gas– liquid	0.08–1.92	0–5	4	4.5	[94]
bifurcation	liquid	0.2–12	5–500	2–8	7–30	[77]
bifurcation	gas– liquid	0.6–5.6	5–200	8	1–10	[9]
split-and-recombine	liquid	3	200	5	1.8	[86]
manifold with fluidic damper	liquid	250	1485	625	1.2	[87]
needle distributor	gas– liquid	superficial velocity fluid 0.018–0.07 ms^−1^	20–250	32–357	0.5–3	[88]
fractal distributor	liquid	2880	1000–100 000	16–64	0.84–3.04	[91]

The fabrication of these distributors and microreactors is not always straightforward due to the many available fabrication techniques and material constraints. The photoreactor material needs to be transparent, which limits the materials. Furthermore, the small dimensions of the channels and shape also limit the fabrication methods.

Advanced fabrication methods, such as 3D printing, micromilling, laser microchanneling, photolithography, etching, hot embossing, and casting were covered in recent review papers [95–97]. The next section will give an overview on the materials that are commonly used for photomicroreactors, together with the advantages and disadvantages.

A popular material for the construction of photomicroreactors is PDMS, and the most common fabrication method to produce PDMS photoreactors consists of two parts: In the first part, a mold is created by 3D printing, soft lithography, milling, or by manually constructing it with objects. The second part consists of casting the PDMS in the mold and curing it. An advantage of PDMS is that it can be bonded to glass, silicon, and itself by treating the surface with oxygen plasma, which oxidizes the surface. This treatment allows the construction of complex geometries by stacking multiple pieces of PDMS. This material was used in the research of Cambié et al. to fabricate a photomicroreactor with 32 parallel channels and a bifurcated flow distributor. They performed the [4 + 2] cycloaddition of 9,10-diphenylanthracene (DPA) with singlet oxygen in acetonitrile [39]. Another example of the fabrication of a photomicroreactor is found in the work of Lamberti et al. in which they produced a microfluidic chip to degrade methylene blue utilizing TiO_2_ as a photocatalyst [98]. Despite the fact that PDMS is utilized to fabricate photomicroreactors, there are some limitations to this material, which includes a limited resistivity against organic solvents and the absorption of small hydrophobic molecules [20,99]. This issue can be solved by coating the surface with a protective coating. Table 3 can be considered to help selecting a suitable material that is compatible with the common solvents used in photochemistry.

**Table 3:** Common solvents used in photochemistry and solvent resistance of PDMS, PMMA and glass.

	solvent resistance^a^		

	PDMS	PMMA	glass

water	+	+	+
acetonitrile	+	0	+
ethanol	0	0	+
tetrahydrofuran	−	−	+
benzene	−	−	+
toluene	−	−	+
acetone	0	−	+

^a^+: resistant, 0: swelling or cracking may occur after long-term exposure, −: immediate damage may occur.

Another polymer that can be used to fabricate photomicroreactors is PMMA. It is noteworthy that PMMA has similar properties and a wider variety of fabrication methods. However, it is not often used for the construction of photochemical reactors. This is due to the lower surface quality, which causes the scattering of the light, making them less transparent [100]. Nevertheless, in the work of Cambié et al., PMMA is used as a waveguide instead of PDMS since more light (40%) is directed to the reaction channel due to the higher refractive index (1.49 and 1.42 for PMMA and PDMS, respectively) [43,101–102]. Recently, a 3D printer was used to fabricate a microchannel from PMMA while incorporating a photoactive monomer, 4,7-distyrene-2,1,3-benzothiadiazole (BTZ), in the reactor structure [103]. This demonstrated that it is possible to directly manufacture a photomicroreactor with 3D printing, allowing a high degree of design freedom. The direct manufacturing of a photomicroreactor has also been shown in the research of Guba et al. [104].

Glass is an excellent material due to the high optical transparence. The wavelength cutoff for quartz and borosilicate is 170 nm and 275 nm, respectively. Furthermore, glass is more resistant to high temperatures and inert to the majority of the solvents used in photochemistry, as shown in Table 3 [105]. Due to the more complex fabrication techniques for glass, it is more expensive and time-consuming to make a glass photomicroreactor. The most common fabrication methods are micromilling, photolithography, and wet etching. Takei at al. used a photomicroreactor with 16 channels created by photolithography and wet etching. The reaction zone was coated with TiO_2_ to catalyze the synthesis of ʟ-pipecolinic acid [16]. Usami et al. utilized a more exotic method to fabricate photoreactors, consisting of partially melting (sintering) glass beads to produce a highly interconnected transparent structure inside a cylinder. The photocatalytic degradation of 4-chlorophenol was performed in this reactor. Due to the high surface-to-volume ratio of the structure (6500 m^−1^), there was a relative increase of 66% in the conversion compared to the reactor without this structure [106].

New techniques emerged to 3D-print complex glass microstructures, which are required to scale up repetitive structures. Kotz et al. presented a 3D printing method to fabricate microchannels with a characteristic length of 20 µm. Moreover, they showed the ability to 3D-print microchannels with a spherical, triangular, trapezoidal, and rectangular cross-section [107]. This allows the fabrication of complex geometries from glass necessary for the intensification of photochemical reactions.

## Conclusion

When combined with a continuous flow mode, micro- and mesostructured photoreactors have the potential to enable efficient and safe operational conditions. As a result, there is a growing interest in micro- and mesostructured photoreactors, leading to a “renaissance” of photochemistry. In this paper, we have discussed the flow and light distribution and mass transport issues of these photoreactors. It was concluded that not only the reactor geometry but also the operational conditions, such as the concentration of the photoactive molecules and the reactants, the residence time, and the light intensity needed to be tuned for an efficient photoreactor operation. A proper coupling of the light source and the reactor geometry is one of the major hurdles of high-throughput photochemistry. It would be good to keep testing new reactor geometries in large scales where the photon flux, mass transfer characteristics, and flow dynamics are characterized. The technical challenges for different structured reactors differ widely. For example, aerosol photoreactors provide a great platform for multiphase reactions. However, if they are going to be used for photooxidations, the lower and upper explosion limits of aerosols should be studied further. For the photomicroreactors, the technical challenges lie in the distribution of light and flow to all units of the microreactors. Much work has been done on the design of flow distributors in two dimensions. However, more research needs to be done on the flow distribution in three dimensions. Different fabrication methods have different challenges. For example, 3D printing is currently limited to certain polymers and metals that are usually not chemically inert, not UV-transparent, or they cannot handle a high temperature and pressure. Utilizing 3D printing of glass in photochemistry would be a great contribution to this field.
